# Multiparametric Monitoring System of Mt. Melbourne Volcano (Victoria Land, Antarctica)

**DOI:** 10.3390/s23177594

**Published:** 2023-09-01

**Authors:** Graziano Larocca, Danilo Contrafatto, Andrea Cannata, Gaetano Giudice

**Affiliations:** 1 Istituto Nazionale di Geofisica e Vulcanologia, Osservatorio Etneo, Piazza Roma 2, 95123 Catania, Italy; graziano.larocca@ingv.it (G.L.); danilo.contrafatto@ingv.it (D.C.); andrea.cannata@unict.it (A.C.); 2 Dipartimento di Scienze Biologiche, Geologiche e Ambientali-Sezione di Scienze della Terra, Università degli Studi di Catania, Corso Italia 57, 95129 Catania, Italy

**Keywords:** sensor network, volcano monitoring, extreme weather conditions, Antarctica

## Abstract

Volcano monitoring is the key approach in mitigating the risks associated with volcanic phenomena. Although Antarctic volcanoes are characterized by remoteness, the 2010 Eyjafjallajökull eruption and the 2022 Hunga eruption have reminded us that even the farthest and/or least-known volcanoes can pose significant hazards to large and distant communities. Hence, it is important to also develop monitoring systems in the Antarctic volcanoes, which involves installing and maintaining multiparametric instrument networks. These tasks are particularly challenging in polar regions as the instruments have to face the most extreme climate on the Earth, characterized by very low temperatures and strong winds. In this work, we describe the multiparametric monitoring system recently deployed on the Melbourne volcano (Victoria Land, Antarctica), consisting of seismic, geochemical and thermal sensors together with powering, transmission and acquisition systems. Particular strategies have been applied to make the monitoring stations efficient despite the extreme weather conditions. Fumarolic ice caves, located on the summit area of the Melbourne volcano, were chosen as installation sites as they are protected places where no storm can damage the instruments and temperatures are close to 0 °C all year round. In addition, the choice of instruments and their operating mode has also been driven by the necessity to reduce energy consumption. Indeed, one of the most complicated tasks in Antarctica is powering a remote instrument year-round. The technological solutions found to implement the monitoring system of the Melbourne volcano and described in this work can help create volcano monitoring infrastructures in other polar environments.

## 1. Introduction

Volcano monitoring is one of the key approaches used to mitigate volcanic risk. Indeed, information from volcano monitoring tasks constitute the only scientifically valid basis for short-term eruption forecasts [1]. Monitoring active volcanoes, the main aims of which are to define the state of the volcano, track its evolution over time and forecast eruptions, is a complex task requiring the acquisition and joint interpretation of a large amount of distinct information and a multiparametric approach [1,2,3]. Indeed, such an approach, gathering information mostly acquired by monitoring instrument networks deployed in the field, is able to track the magma migration in depth, as well as the evolution in time of its features. Hence, the development of volcanic monitoring systems requires the creation of a multiparametric network composed of different types of instruments deployed on the volcanoes. In particular, the main instruments that are installed on volcanoes for monitoring purposes are seismometers (able to record the different types of volcano seismicity), ground deformation stations (mainly GNSS and tiltmeters, to measure even very slight deformation of the volcanoes), infrasonic microphones (to record elastic energy releases in the atmosphere), webcams and in situ or scanning gas sensor networks (to record variations in time of both the composition and amount of gas released by the volcano) [2]. In addition, remote volcano monitoring systems are being used more and more and are also rapidly improving [2,4]. However, to date, in situ monitoring is still the best strategy to assess the state of activity of a volcano and its changes over time.

Active volcanoes are spread all over the world and are also in remote areas such as the Antarctic continent, where the installation and maintenance of scientific instruments can be really challenging. Such instruments must face the most extreme climate on the Earth, characterized by very low temperatures and strong winds, typical of katabatic wind regimes [5,6]. The most complicated task is powering a remote instrument year-round because of many factors [5]: (i) solar radiation changes during the year from whole days of sun during the Antarctic summers to complete darkness during winters; (ii) strong winds, often unpredictable in duration and strength, which can cause severe issues when wind turbines are used as power sources; (iii) the cold temperatures, which decrease the capacity of most batteries. An interesting solution to maintain continuous power for scientific instruments was recently proposed by [7], who suggested to use the harvesting of thermal energy collected near the summit crater of the Erebus volcano.

**Figure 1:**
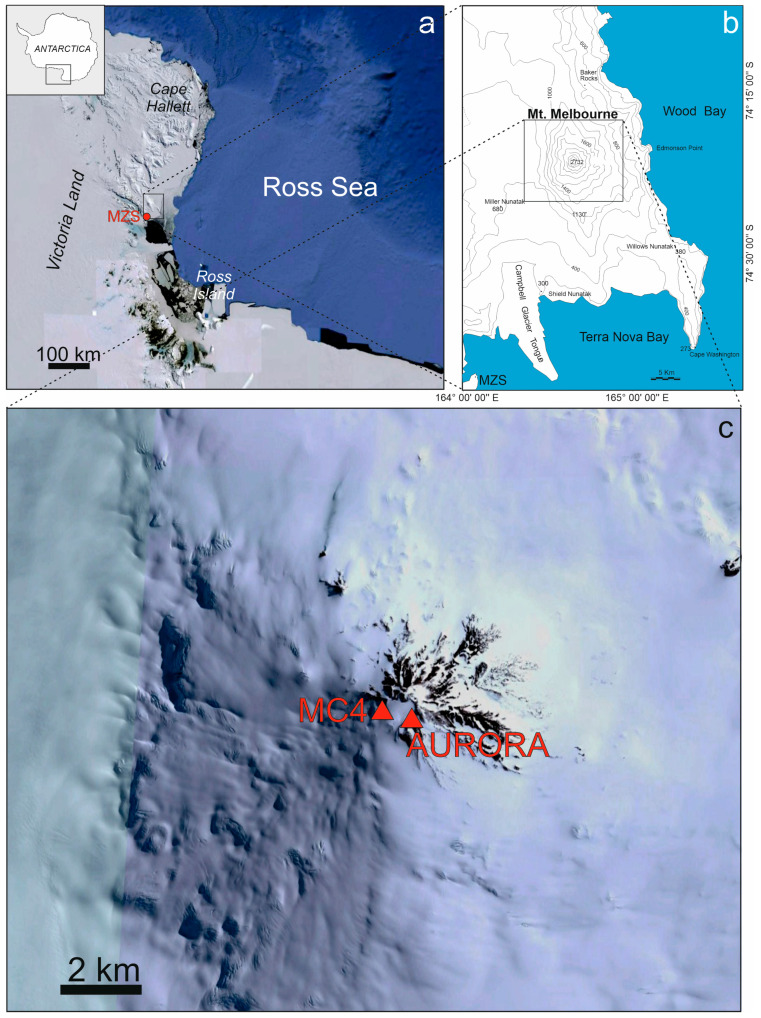
(**a**) Aerial image of Ross Sea area from Google Earth with the location of the installation site of Mt. Melbourne volcano (black square) and Mario Zucchelli station (MZS, red dot). The inset in the upper left corner shows the location of the Ross Sea area in the Antarctic continent. (**b**) Map of the Mount Melbourne area. (**c**) Aerial image of Mt. Melbourne volcano from Google Earth with the location of two fumarolic ice caves used for the monitoring station installations (orange triangle).

The most recent volcanic activity in Antarctica is associated with the West Antarctic Rift system and the Antarctic Peninsula region [8]. The former is one of the largest areas of crustal stretching in the world and runs from the base of the Antarctic Peninsula in the Weddell Sea to the Ross Sea Embayment in northern Victoria Land [9]. Mount Melbourne (Figure 1), located along the western shoulder of the West Antarctic Rift system in north Victoria Land and standing at an elevation of 2732 m above sea level, is one of the largest active volcanoes of Antarctica. It is approximately 42 km away from the Italian Mario Zucchelli station (MZS) and close to other scientific stations such as Jang Bogo and Gondwana. This volcano is mostly concealed beneath ice, with the exception of certain peripheral regions such as Shield Nunatak, Edmonson Point and Baker Rocks. Additionally, on its summit and upper slopes, Mount Melbourne features a multitude of scoria cones, lava domes, highly viscous lava flows and expanses of exposed lava fields [8,10]. The summit region is characterized by a roughly one-kilometer-wide crater filled with snow, and it is encircled by several scoria cones that have been responsible for the majority of the phonolithic black bombs and scoria fragments [10]. The last eruption of Mount Melbourne is likely to have occurred around 1892 CE [10,11]. Since the last eruptions were explosive and associated with evolved magma compositions, sub-Plinian/Plinian explosive activity could potentially occur in the future [12]. Moreover, the presence of ice enhances the risk of hydrovolcanic eruptions, which could turn small-volume eruptions into highly explosive ash-forming events due to magma–water interactions [13,14]. Hence, the institution of a monitoring network would be the only way to mitigate the risk associated with future eruptions of the Melbourne volcano for the Antarctic communities that are its immediate neighbors, but also to mitigate possible serious problems for the other Antarctic stations which are logistically linked to the Melbourne area (i.e., McMurdo and Scott bases).

In this work, we describe the multiparametric monitoring system recently installed on the Melbourne volcano, focusing on the strategies applied to make the stations work for most of the year despite the extreme Antarctic meteorological conditions and thus allowing the acquisition of seismic, geochemical and thermal data. It is worth noting that only a few attempts to create geophysical monitoring systems were made in the past on Mount Melbourne [15], and almost no attempts were made regarding geochemical monitoring systems. In addition, unlike the Melbourne observatory of the 1990s, in the new observatory the data are acquired, transmitted and analyzed in near real time. Among the strategies applied in the new multiparametric monitoring system, of particular importance are the choice of the fumarolic ice caves as installation sites for most of the monitoring devices and the special measures to power the stations and reduce the energy consumption, such as the use of large-energy-capacity accumulators and particular operating mode settings of the instruments.

## 2. Ice Caves and Installation Sites

The site selection for permanent station installation is a particularly important task in Antarctica. The fumarolic ice caves (hereafter referred to as FICs), explored and mapped in the framework of the research projects ICE-VOLC and MIMIC [10], represent a particularly protected place suited to host the equipment necessary for continuous volcano monitoring.

The interaction between permanent snow/ice layers and hot fumarolic gas causes the formation of FICs, and only a few environments around the world host this particular kind of cave, like active volcanoes in the USA (Cascade Volcanoes), Iceland, Kamchatka and Antarctica [16,17]. Mount Melbourne is characterized by fumaroles fed by volcanic fluids [18]. In these areas, the surface expression of the degassing zone associated with the fumarolic fields is provided by both FICs and ice towers. The hot gas and steam, which escape from the volcanic surface, melt the lower layers of ice and snow, creating underground environments often composed of an intricate network of rooms and passages: the FICs.

**Figure 2:**
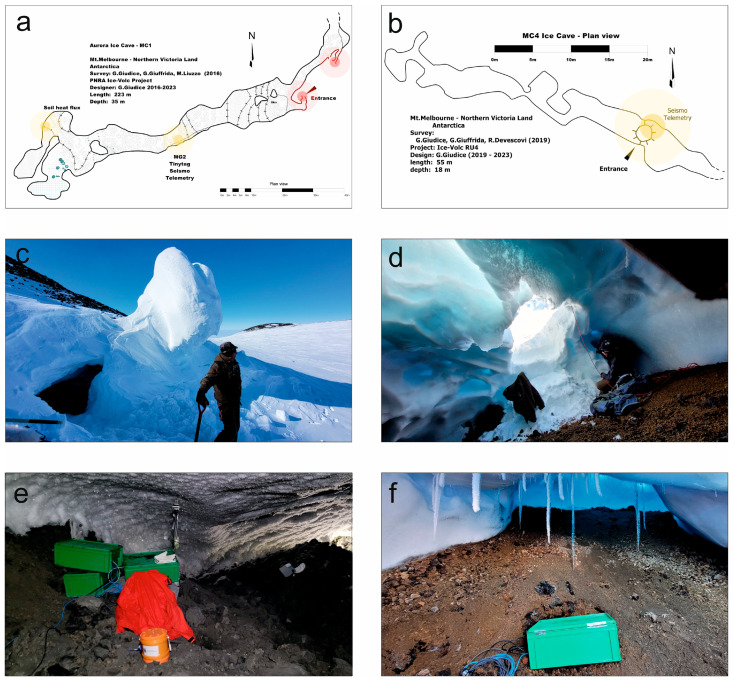
(**a**,**b**) Maps of the Aurora and MC4 ice caves, respectively. (**c**,**d**) Pictures showing the entrance of the Aurora and MC4 ice caves, respectively. (**e**,**f**) Pictures showing rooms of the Aurora and MC4 ice caves, respectively, where instrumentation was installed.

The degassing phenomenon through the FICs also gives rise to typical chimney-like structures, located up the slope and visible from the external ice field, called fumarolic ice towers (FITs). The temperatures inside the FICs are close to 0 °C all year round and, in addition, despite the external adverse meteorological conditions, no storm can damage the instruments. Two of the FICs explored on Melbourne have been selected to deploy instruments: Aurora (also indicated as MC1) and MC4, located at the opposite edges of the caldera (Figure 1 and Figure 2). Aurora is the first FIC explored on the top of Melbourne and has two FITs: the lower one is used to access the cave (Figure 1 and Figure 2a,c,e). Concerning the structure of the Aurora FIC, from the entrance there are a couple of relatively small passages (often less than 1 m wide) with pyroclasts of different sizes (from ash to lapilli and bombs) on the soil, while the snow–ice walls are changing in size due to variations in heat flux and thus to differences in thermal erosion. After, there is a steep slope of about 30 m in a large tunnel (around 2 × 4 m wide), equipped with a rope to ease travel, leading to the monitoring site, which is a gallery around 5 m wide and 1.5 m high in a sector of the cave convenient to measure the gas fluxes. A seismometer, a multi-gas and meteo station, a CO_2_ accumulation chamber system and a temperature logger are deployed there. Continuing the path, after about 30 m a smaller gallery leads to a wide room, where two temperature sensor loggers, buried in the soil, are deployed. The MC4 cave (Figure 1 and Figure 2b,d,f) shows a big FIT on the upper part, sometimes used to enter the cave by a metallic flexible ladder, whereas on other occasions it is possible to enter directly into the gallery from the side wall without ropes or ladders. Although the cave’s length is around 60 m, the monitoring site is close to the entrance to ease the maintenance of the deployed devices. In this case, just a seismometer and a temporary radio transmission system are installed.

## 3. Monitoring System

**Figure 3:**
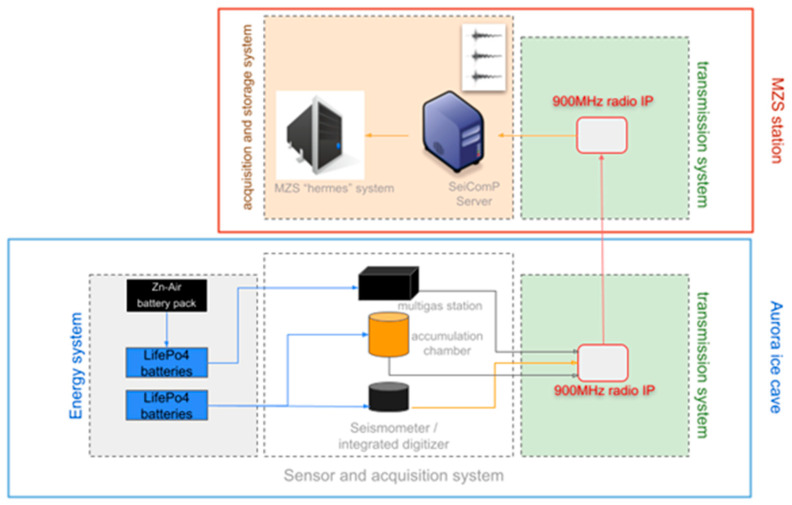
Sketch of the monitoring system composed of different parts: sensor and acquisition system; power and energy management system; transmission system and acquisition system at Mario Zucchelli station.

The monitoring system is composed of different parts: seismic stations, geochemical and thermal stations, a power and energy management system and a transmission and acquisition system (Figure 3).

### 3.1. Seismic Stations

Seismic sensors in a polar volcanic environment should have some fundamental characteristics, which are as follows:

- Inexpensive in terms of power consumption.
- Have operating temperatures compatible with extreme temperatures. Inside the ice caves the average annual temperature is around zero degrees; therefore, sensors that have an operating temperature range down to −20 °C are more than sufficient. In outdoor installations, temperatures drop significantly in winter, even reaching −60 °C. In this case, it is advisable to rely on sensors specially designed for polar areas with guaranteed operating temperatures of at least −55 °C. Experience in the field has also taught us that some sensors that are not guaranteed at temperatures below −20 °C have given excellent results at much more extreme temperatures, such as the Nanometrics Trillium, widely used in Antarctica by multiple scientific groups.
- The quality of a seismic transducer is also evaluated on the basis of its self-noise, i.e., the noise it produces by itself without any natural or artificial vibration applied. The internal noise produced by the same instrumentation can be a limit to the detection of signals attributable to microseismicity, especially when it is necessary to monitor a quiescent volcano such as Melbourne, where it is important to be able to investigate for signs of volcanic activity which can be energetically very limited. It is therefore important to analyze these data released by the manufacturer, which are very often represented by a frequency response of the sensitivity with reference curves called NLMN and NHMN, Peterson’s low noise and high noise models, respectively.
- A flat sensor response over a wide frequency range (at least up to period of 40 s), to be able to properly record the long-period seismicity typical of volcano seismicity.

**Figure 4:**
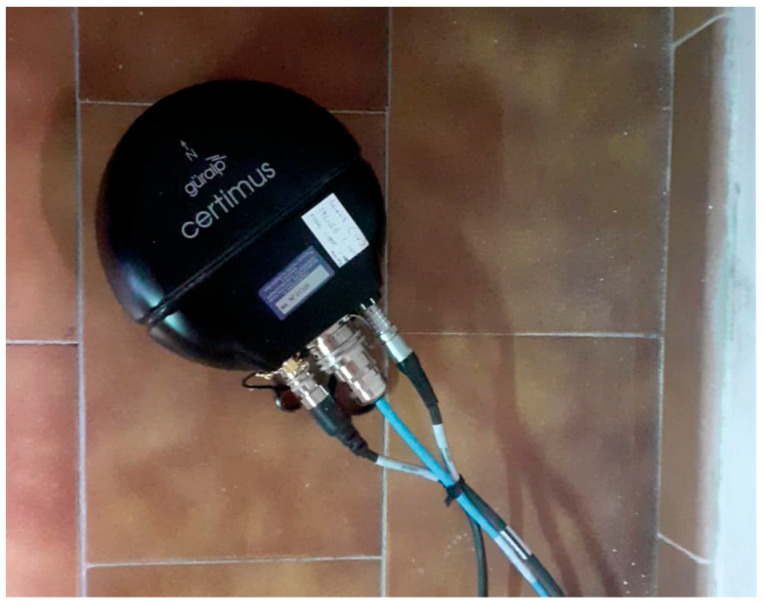
Guralp Certimus digital seismic sensor during a laboratory test.

During the Antarctic campaign in November-December 2022, two seismic stations consisting of Guralp Certimus were installed (Figure 4). This instrument is a 3C broadband velocimetric sensor with an embedded digitizer. This model was chosen because of several features. First of all, Guralp Certimus combines a triaxial broadband sensor with a flat band-pass up to 100 Hz and a lower frequency corner of 0.0083 Hz (120 s period). This bandwidth guarantees us a linearity of operation necessary to also record the long-period (LP) and very-long-period (VLP) seismicity typical of most active volcanoes.

The embedded digitizer, called “Minimus”, is a very compact instrument (175 mm diameter and 95 mm total height). In addition, the system is protected by an anodized aluminum casing that protects the internal electronics from water, allowing installations in various difficult environmental contexts. Furthermore, the system is equipped with a MEMS-type accelerometer that facilitates and speeds up installation, allowing the instrument to be positioned even if not level to the ground and with a very wide range of tilt angles. The model we use is not equipped with a liquid crystal display as in the standard model to allow its burial and the use in environments with extreme temperatures, as well as to guarantee significant energy saving in terms of current consumption. Regarding this, current measurements were carried out in the laboratory, detecting an average consumption of about 150 mA with 12.6 V (1.90 W) in “deploying normal” mode (ethernet port and active GPS receiver).

Another characteristic evaluated in the choice of the type of seismic stations is the operating temperature, guaranteed by the manufacturer, ranging from −40 °C to 85 °C. Data synchronization is performed by an integrated GNSS receiver connected to an external antenna which optimizes the reception of the Navstar (GPS), GLONASS, Beidou and Galileo constellations. Certimus is equipped with a 64 GB microSD card, which allows the continuous recording of a velocity signal sampled at 100 Hz for more than a year. The microSD is hot-swappable and therefore removable from the external case at any time for immediate data backup or replacement with a blank microSD. The two stations installed on the Melbourne volcano were configured to sample the velocimetric signal at a frequency of 100 Hz, with “deploying normal” or “GPS and LAN Power Save” operating mode (see Section “Power and energy management system” for details). Each station was correctly oriented towards geographic north before being definitively buried.

Concerning the instrument calibration, for the entire time period when they will be used in the field, laboratory calibrations of the velocity sensor are not envisaged, as the manufacturer does not prescribe periodic laboratory recalibration. In fact, in the absence of particular problems, the sensor response should be constant.

### 3.2. Geochemical and Thermal Stations

**Figure 5:**
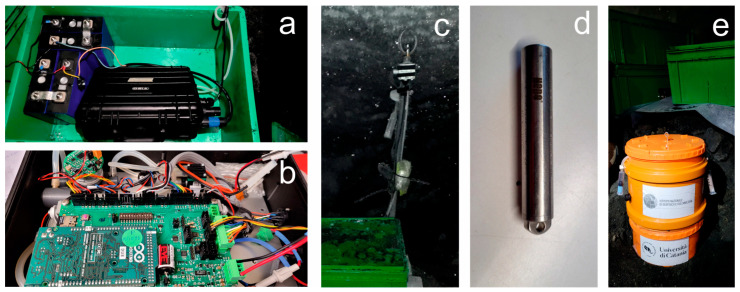
(**a**) Picture showing the multi-gas station and the battery pack feeding it. (**b**) Detail of the electronic board of the multi-gas in (**a**). (**c**) Air thermal sensor and (**d**) soil thermal sensor. (**e**) Accumulation chamber system.

Since 2016, inside the Aurora FIC a geochemical automatic station has been deployed around 50 m from the entrance (Figure 2a,c,e). This multi-gas station (called MG2) measures the main gas components, especially CO_2_ and H_2_O concentration, wind speed and pressure and temperature, in order to evaluate changes in gas composition/flux as well as in heat flux (Figure 5a,b). This information is very important in detecting the possible onset of unrest phases. A temperature datalogger (Tinytag Gemini) device is also located in the same place to assure autonomous temperature records (Figure 5c), while since 2017 a couple of temperature sensor loggers have been installed 20 and 60 cm underground (Hobo devices; Figure 5d) to estimate the heat flux from soil around 100 m from the entrance. As for the wind speed sensor, it is worth noting that we also tested a couple of ultrasonic sensors, but they only worked for a few days and then they became unresponsive, maybe due to the permanent layer of frozen condensate that formed after a brief interval from the deployment. Hence, a new hot wire thermo-anemometric sensor was adopted, capable of measuring the low-speed winds typical of the Aurora ice cave.

In 2022, a second geochemical station consisting of a prototype of a static accumulation chamber system without moving parts was deployed near the first station to evaluate the CO_2_ flux from soil (Figure 5e).

Concerning the installed thermal and geochemical sensors, the main features are the following:

- Soil temperature sensor: range −40 °C to 125 °C; accuracy ±0.22 °C; resolution 0.05 °C;
- Air temperature sensor: range −40 °C to +125 °C (−40° F to +257° F); accuracy 0.4 °C; resolution 0.05 °C or better;
- Electrochemical sensor: range 50 ppm; resolution 0.5 ppm; repeatability 1% of signal (and 2% calibration gas tolerance);
- NDIR (Non-Dispersive Infrared) CO_2_ spectrometer: 10,000 ppm range; +/− 2% range accuracy (and 2% calibration gas tolerance);
- Humidity: range 0 to 100% rh; accuracy (5 to 95% rh at 10 to 40 °C) ±2% rh;
- Pressure: range 200 to 2500 mBar; accuracy ±1.5%;
- Wind speed: resolution 0.01 m/s; accuracy ±0.05 m/s.

These are also the features we suggest for thermal and geochemical sensors used in volcano investigations. In addition, it is worth noting that these sensors must not possess particular characteristics in terms of operating temperature, as the conditions inside the caves are within the operational characteristics of all the used geochemical and thermal sensors.

Finally, to calculate the CO_2_ fluxes from soil, we used the RANSAC linear algorithm to process the CO_2_ concentration variation over time inside the accumulation chamber (see [19] for further details). Concerning the instrument calibration, we calibrated the sensors with standard reference gases in Italy before their deployment. We change the electrochemical sensors with new calibrated ones on every Antarctic campaign, so on average every one to two years.

### 3.3. Power and Energy Management System

The power supply system has been designed considering some fundamental factors such as the operating temperatures, the effective duration of activation of the instrumentation and the installation context.

For the geochemical and seismic stations installed on Mt. Melbourne, the context is offered by the FICs, which imply the absence of sunlight and ambient temperatures showing small changes and remaining for most of the year around 0 °C. Furthermore, the stations are connected via a serial RS232 interface to a radio for data transmission that is active for a relatively short time (a few months). An exception is the radio repeater, which is positioned outside the FICs with the possibility of exploiting solar energy for recharging. The temperatures in this case are much more variable and extreme, with recorded values of even <−35 °C during the test period (about 3 weeks from November to December).

**Figure 6:**
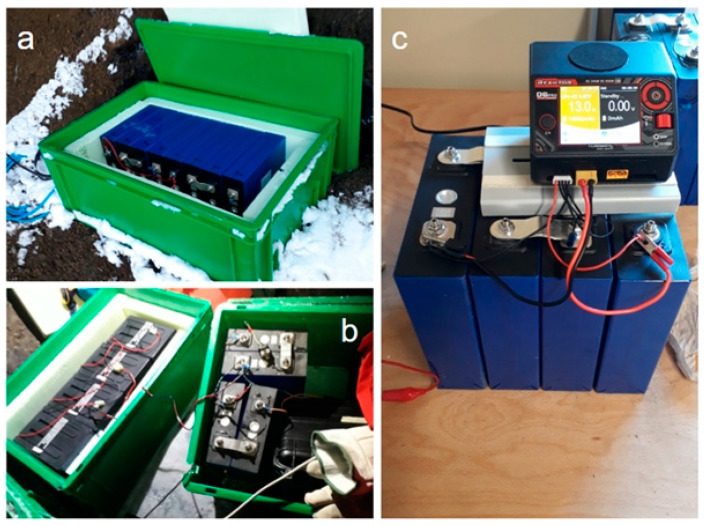
Picture showing batteries and controllers: (**a**) LiFePO4 battery pack for seismometer, 2 × 176 Ah 13.2 V; (**b**) 2 × 175 Ah 18 V Zn-Air battery pack as trickle charger for 1 × 176 Ah 13.2 V LiFePO4 pack, for geochemical station; (**c**) 1 × 176 Ah 13.2 V LiFePO4 battery under charge with automatic high-power battery charger and cell balancer.

On the basis of all these factors, a power system made up only of large-energy-capacity accumulators, without recharging, was chosen. The power system was therefore composed of blocks of 176 Ah rechargeable lithium batteries (called LiFePO4), each of which is composed of four 3.6 V lithium cells (Figure 6). Lithium batteries have major advantages over, for example, lead-acid ones: they are much lighter and easier to transport even in uncomfortable conditions such as inside caves and they maintain their nominal capacity even at very low temperatures.

Another key parameter to consider when designing a power system is the power consumption with the load activated: while the seismometers are continuously activated, the geochemical stations are activated just twice per day for about 30 min each time, so most of time they are in stand-by state with a minimum current consumption with respect to the seismometers.

Concerning the geochemical multi-gas station, a main block of 176 Ah rechargeable lithium batteries was adopted, with a trickle charging system composed of a parallel 2 × 175 Ah 18 V (series of 2 × 9 V elements) Zn-Air battery pack and a custom-made low-power current regulator, so that a complete year of autonomy is expected. Zn-Air batteries are cheap, easy to manage and safe to transport, even during flights, but are able to guarantee only low-level continuous current, so they were adopted just to recharge the main rechargeable lithium batteries during the long stand-by periods of the geochemical stations.

The electrical current measurements carried out in the laboratory have shown that the seismic stations consume, in normal operating conditions, about 150–160 mA, while for radios in continuous transmission we have a current consumption of a further 170 mA. Using these values and assuming the use of the nominal capacity of the 176 Ah lithium battery blocks, in theory we could obtain an autonomy from the system made up of batteries, Certimus and radios in continuous transmission of approximately 502 h, that is, almost 21 days. The considerations just made were then confirmed in the field; the seismic station in the Aurora FIC was put into operation with transmission starting from 12 November until the beginning of December without changing batteries, i.e., about 20 days, without any problems.

The Mario Zucchelli station is closed from mid-February to mid-October, and during this time of the year there are no staff who can deal with services like the maintenance of the radio repeater on the summit of Melbourne. This means that when the Mario Zucchelli station is closed, data transmission is not feasible and thus the use of the ethernet port of the seismic station becomes an energy waste. For this reason, in February the operating mode of the seismic stations was turned from “deploying normal” to “GPS and LAN Power Save”, permitting to save almost 50% of energy. Current consumption measurements performed in the Mario Zucchelli station laboratory demonstrate that in this operating mode, most of the time Certimus uses only 75 mA, with 12.6 V of supply voltage (<1 W power consumption). This operating mode turns off the ethernet port and the GPS receiver, but the latter is activated periodically for a few minutes to maintain synchronization of the internal real-time clock and of the acquired signal. When the GPS receiver is activated, we measured 100 mA of current consumption. Overall, we used this operating mode to try to record continuous seismic signals and store them in the station internal memory from February to October (autumn and part of winter season), effectively almost doubling the battery life. Obviously, this configuration does not allow us to monitor or analyze seismic signals in real or near real time during this time interval.

Furthermore, when we replaced the exhausted batteries with other charged batteries, we added another block of 176 Ah batteries, connected in parallel to the previous one, obtaining a single 352 Ah storage system. Under these conditions we expect that the seismometers will run for about 6 to 7 months, while the geochemical station might last for more than one year.

### 3.4. Transmission and Acquisition System

One of the biggest challenges faced during the Antarctic campaign was the data transmission in real time from the Melbourne volcano. The two stations in the FICs were equipped with radios in the 900 MHz frequency spectrum, which allowed the transmission of seismic and geochemical data and ensured great reliability. The presence of ice and snow between the transmission point inside the caves (thicknesses up to 10 m) and the external repeater or the receiver in the MZS was not an issue, and only a fine alignment phase of the antennas in the caves was necessary.

In particular, FreeWave© Zumlink radios were used, a type of radio manufactured in the United States that ensures an excellent degree of robustness and reliability even in extreme environments. The ZumLink radios operate in the unlicensed 900 MHz spectrum and utilize Frequency Hopping Spread Spectrum (FHSS) technology for cybersecure data transfer with RF link rates up to 4 Mbps. Moreover, the transmission can reach distances between the transmitter and receiver of about 97 km with a clear line of sight and the energy consumption is relatively low, with 377 mA in transmission and 153 mA in receiving. Also, these radios operate in temperatures from −40 °C to +75 °C, useful for the extreme environment where they were used. In our case, a total of 4 radios were used to transmit data from two FICs and receive them at the Mario Zucchelli base.

To improve the transmission and reception of the signals, Pasternack yagi antennas were used together with these radios, which guaranteed greater directionality of the transmitted and received radio signal with a gain of 6 dB in the frequency range from 824 to 960 MHz. With a very compact shape and formed by a dipole and three director elements, a yagi antenna is very robust and resistant even to the strong winds present in the Antarctic environment.

**Figure 7:**
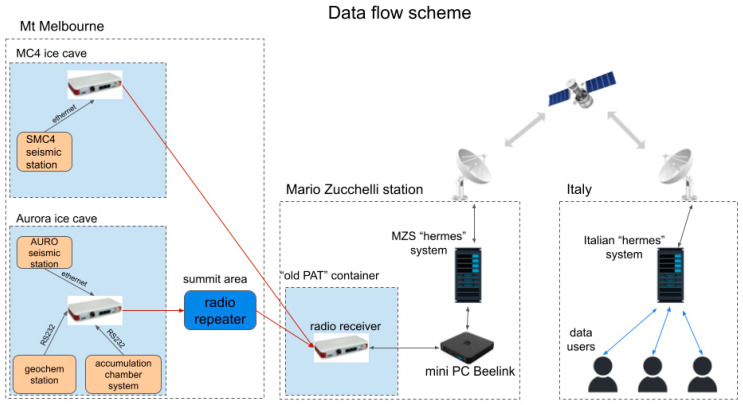
Sketch of the transmission and acquisition system.

As can be seen in the sketch in Figure 7, a receiving radio was installed in the Mario Zucchelli station inside a container called “old PAT”, where it was possible to obtain a power supply, a connection to the LAN network of the base and a comfortable passage of the RF cable to the outside, where the yagi antenna was placed pointing towards the Melbourne volcano and positioned with vertical polarity. The distance covered by the radio link between the reception point and the transmission points on the top of Melbourne is around 42 km. The seismic station positioned inside the MC4 FIC has a suitable position for a direct point-to-point transmission, as the Mario Zucchelli station is in radio visibility. In fact, the station is located at the entrance to the ice cave, and it was thus possible to connect the yagi antenna, using an RF cable of suitable length, by fixing it to a temporary wooden pole near the entrance.

Instead, as regards the sensors installed inside the Aurora FIC, it is not possible to use a direct radio link to reach the receiver at the Mario Zucchelli station. For this reason, it was necessary to use a third radio, which acted as a radio repeater for the signals coming from Aurora. The radio repeater system, consisting of a FreeWave radio, a yagi antenna and lithium batteries, was placed inside a waterproof box. The yagi antenna was placed in vertical polarization. Connection tests were carried out with the base and the radio inside the Aurora FIC to determine a point at the top of the crater, above the Aurora FIC, which allowed us to obtain an optimal radio link. The box containing the radio repeater was suitably positioned to “point” the yagi antenna in the direction of the Mario Zucchelli station. The installation gave excellent results, as the box made it possible to protect the repeater instrumentation from the winds and the extreme environmental conditions on the top of the volcano, allowing for total continuity of the radio link.

The radio is capable of carrying standard protocols and IPs over Ethernet and serial ports (PPP), so when you connect the receiver to the base LAN, the other radio-connected points on Melbourne are also part of the base LAN, they have a single static IP address assigned to them and they can be reached and configured remotely via the web interface.

The data transmitted by the stations, located in the Melbourne FICs, are received by the radio gateway placed in “old PAT” and flow into the local computer network of the Mario Zucchelli base. A mini PC active in the laboratory then takes care of the acquisition and buffering of both seismic and geochemical data. In particular, a Beelink^®^ mini PC model BT3 with an Intel Atom x5-Z8350 processor, 4 GB RAM memory and 64 GB internal storage was installed. It is also equipped with an HDMI port, a VGA port used to connect a monitor, a 1 Gbps LAN Ethernet port, 4 USB ports and an SD card reader. This hardware system was chosen because the current consumption is moderate, and this is particularly important in view of a definitive installation inside the room called “PAT” of the Mario Zucchelli base, where the power is guaranteed all year long, even during the Antarctic winters. The operating system of the mini PC is a Linux Ubuntu 22.04 LTS, which ensures excellent productivity and stability, allowing, among other things, the installation of the software useful for seismic data acquisition, called SeisComP version 4. SeisComP is a seismology software for data acquisition, processing, distribution and interactive analysis developed by the GEOFON Program at the Helmholtz Center Potsdam GFZ German Research Center for Geosciences and Gempa GmbH [20].

SeisComP is one of the most distributed software for real-time seismological acquisition and internet data exchange. This is free software distributed for non-commercial use. By using SeisComP, we had the possibility to remotely manage the data request to the Certimus stations using the Seedlink transmission protocol, a robust transmission protocol supporting TCP/IPs. With this protocol, software clients that connect to remote stations to request a continuous real-time data stream can connect and disconnect without losing data, as the lost data can be recovered as long as the data exist in the remote station’s buffer. This feature makes it effective for managing the acquisition of stations installed on the Melbourne volcano, as the radio transmission can be discontinuous over time due to various temporary problems producing data gaps. A software specifically written in python called DLServer and installed inside the mini PC was used to manage data coming from the geochemical stations. The geochemical and seismic data are therefore buffered inside the mini PC for a certain period and then overwritten by the newest ones. Once buffered, the data must be sent to Italy to carry out constant remote monitoring of the Melbourne volcano. To perform this task, ENEA has set up and manages two archive servers suitably configured to carry out storage operations and the automated distribution of the archives located at the base in Antarctica and in Italy.

This system is called “hermes” and is schematically composed of a server installed at the Mario Zucchelli base and another located at the ENEA headquarters in Casaccia (Italy; Figure 7). The system performs an automatic check to see if data are found in the folders of the various accounts, and when it finds any, it transfers the data to the other server located in Italy, taking advantage of the limited satellite bandwidth available at the base in Antarctica.

Adapting to the system described, we created a Linux bash script to automatically transfer daily miniseed files and geochemical data to the MZS “hermes” system, thus also allowing the automatic transfer of data to Italy. In particular, the Linux utility command rsync was used for efficient data transmission and to ensure mirroring of the miniseed and geochemical files created starting from the real-time data stream coming from the remote stations. The script was run once a day using the Linux cron service to schedule it.

## 4. Discussion on Recorded Data

Several analyses were performed on the data recorded by the monitoring system to extract valuable information from the raw signals.

As for the seismic data, the main purpose of these analyses is to characterize both long-lasting signals and amplitude transients. As for the former, the signals are mostly derived from the ocean–solid Earth interaction (the so called microseism, the most continuous and omnipresent seismic signal on Earth [21]) or from fluid dynamics within the volcano plumbing system (volcanic tremor; [22]). Concerning the amplitude transients, several types of events can be recorded, such as ice-quakes, earthquakes, long-period events and very-long-period events. Hence, to automatically identify amplitude transients and define their duration, the short-time average/long-time average (STA/LTA; [23]) algorithm, based on the comparison of short-term average amplitude (STA) and long-term average amplitude (LTA) of the signals, is used. This algorithm is applied in several frequency bands, appropriately choosing the length of the STA and LTA windows (see [23] for details) to identify transients with different spectral contents. To characterize the frequency content of the seismic signals in the case of both long-lasting signals and amplitude transients, spectra and spectrograms calculated by the Fast Fourier Transform (FFT; [24]) algorithm are obtained. The spectral information is particularly important to try to understand the source mechanism of the recorded signals. For instance, in case of long-lasting signals, low frequencies (generally below 0.5–1.0 Hz; [25]) are characteristic of the microseism, while higher frequencies can be indicative of a tremor with volcanic origin [22]. The amplitude of long-lasting signals is estimated by different methods, such as the Real-time Seismic Amplitude Measurement (RSAM; [26]) and the root mean squared (RMS) amplitude [27], while the amplitude of the transients can be also estimated by using the peak-to-peak amplitude parameter. In addition, when the seismic network is improved in terms of the quantity of sensors and their spatial coverage, a location analysis of the amplitude transients is also performed. The determination of the source depth is an important parameter used to discriminate the glacial sources, generally very shallow, from the volcanic ones, generally deeper.

Concerning the geochemical and thermal data, the aim is to monitor the variations in the relative abundance of the main gases, their mass fluxes and the possible increases in soil and ambient temperature inside the ice caves; hence, the analyses often involve visualization of the time series and searches for particular patterns. Concerning CO_2_ fluxes, they receive more attention in volcanic surveillance, as they are considered as a good indicator of the migration of magmatic volatiles [28]. Thus, we focus on the increasing trends in CO_2_ fluxes that could suggest unrest phenomena. It is also easy to understand that detecting an increasing trend in temperatures in soil and air inside the ice caves can be directly related to volcanic unrest. Also, in the case of thermal data, we look for variations in time in the thermal time series to obtain information about the beginning of unrest phases. Another important piece of information is SO_2_ concentration, which is at the moment undetectable, because an increase in this parameter would represent a clear indication of unrest. Regarding these gas fluxes and thermal measurements, FICs located on the volcano summit are suitable places where these parameters can be estimated, as the caves act as warm gas integrators, which allows the detection of eventual anomalies. Indeed, even if the single fumarole contribution can be relatively small, the cave itself collects the outputs of many fumarolic areas along the tunnel slope, increasing the sensitivity with respect to anomalies.

The results of these analyses performed on all the acquired signals for some years are needed to define a background or baseline behavior. Once this is established, the thresholds beyond which a volcanic unrest is declared will be defined.

**Figure 8:**
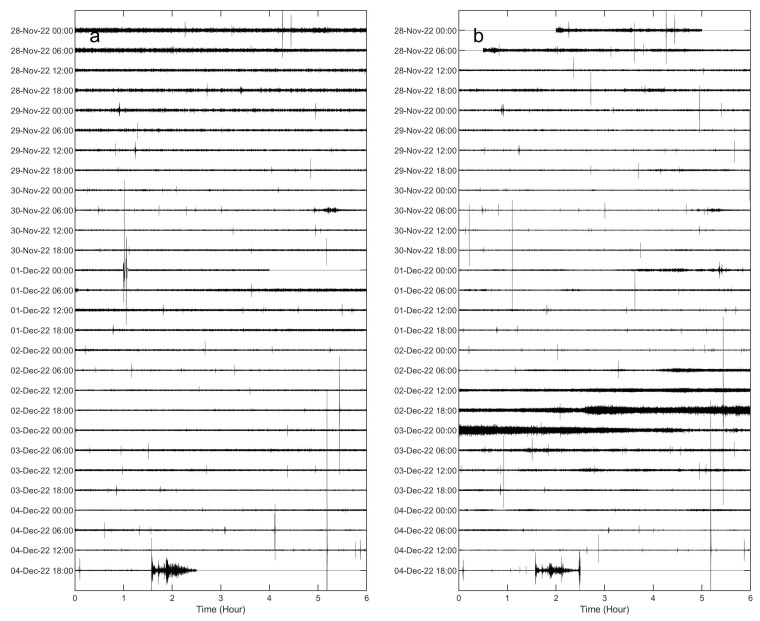
Example of signals recorded by the vertical component of the seismic stations AURO (**a**) and SMC4 (**b**) during the interval 28 November–4 December 2022.

**Figure 9:**
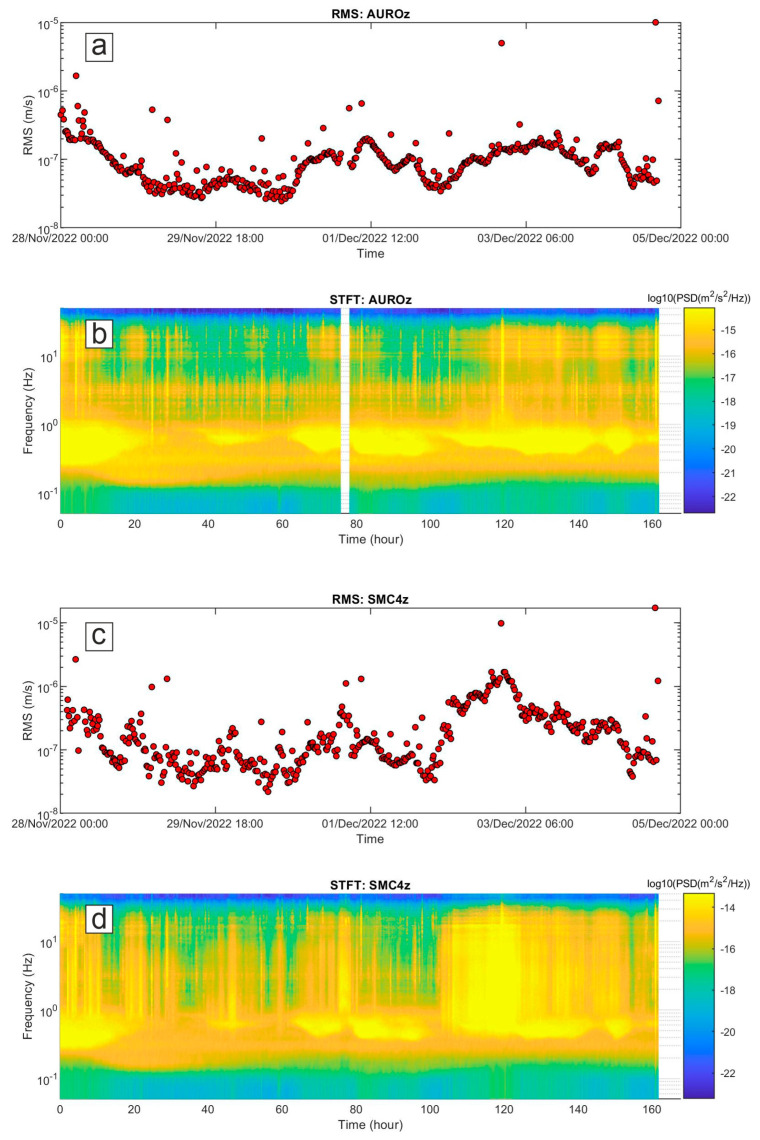
Time series of root mean squared (RMS) amplitude of the vertical component of the seismic stations AURO (**a**) and SMC4 (**c**) during the interval 28 November–4 December 2022, and corresponding spectrograms (**b**,**d**). The RMS amplitude values were computed on the vertical component of the seismic signal filtered in the band 0.1–40 Hz. As for the spectrograms, each spectrum representing a 10 min long seismic signal was calculated by applying Welch’s method [38] with time windows of 81.92 s. The spectra, thus obtained, were gathered with time on the x-axis, frequency on the y-axis and the log10 of the PSD indicated by a color scale.

**Figure 10:**
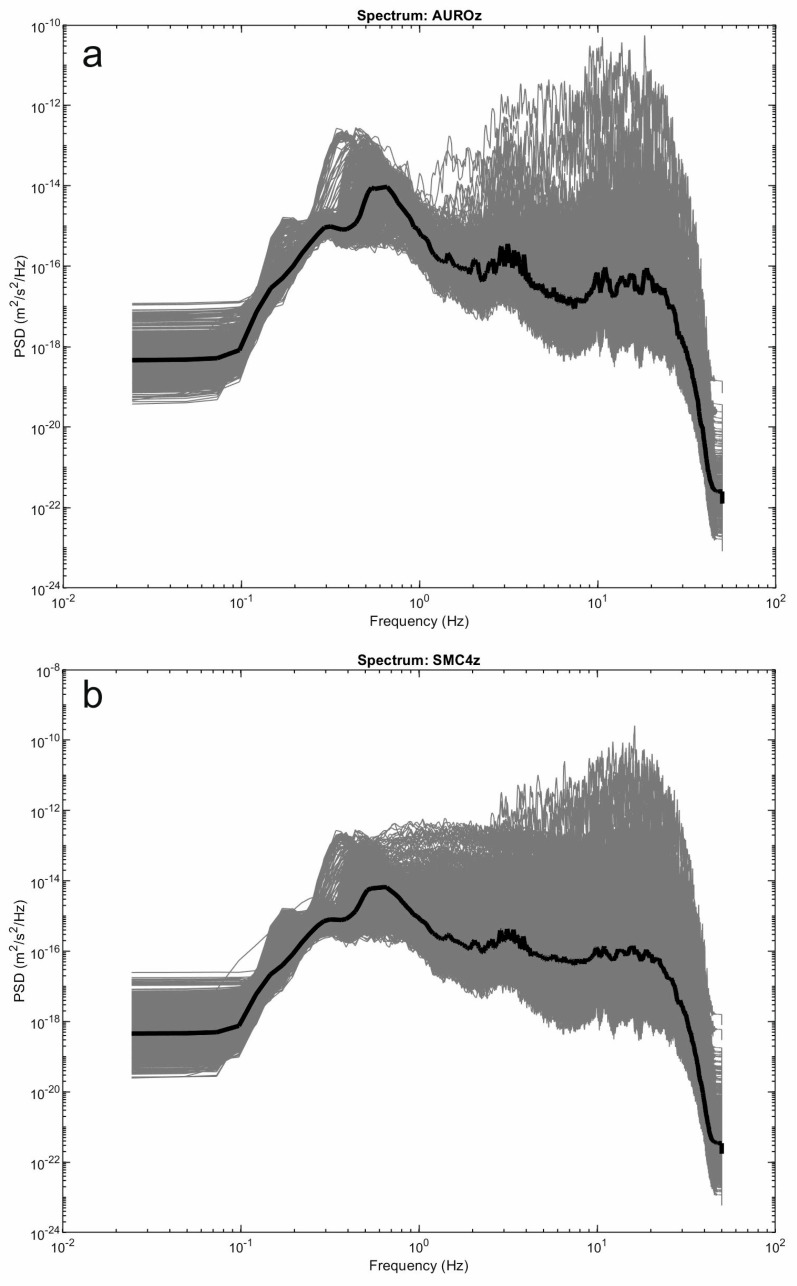
Spectra calculated on 10 min long windows of the signal recorded by the vertical component of the seismic stations AURO (**a**) and SMC4 (**b**) during the interval 28 November–4 December 2022 (grey lines) and corresponding median spectra (bold black lines).

Here, we show examples of data collected by the multiparametric stations of Melbourne, as well as of the results of some of the aforementioned analyses. Concerning seismic signals, data recorded during 28 November–4 December 2022 were analyzed in terms of both continuous signals and amplitude transients (see helicorders from the vertical component of AURO and SMC4 stations, shown in Figure 8). Spectrograms and spectra in Figure 9 and Figure 10 exhibit that most of the energy is recorded in the band of 0.1–1.0 Hz, related to the microseism [25]. In particular, the abovementioned band comprises secondary and short-period secondary microseisms that are likely due to the interactions of sea waves of equal frequency travelling in opposite directions and generating standing gravity waves (secondary microseism; [29]) or to sources generally linked to the local sea state and wave activity and influenced by local winds (short-period secondary microseism; [30]). The temporal variations in seismic amplitude, clearly observed in both the spectrogram and the time series of root mean squared (RMS) amplitudes, mostly reflect the energy modulation of the microseism, generally due to sea wave activity (e.g., [31,32]). It has to be noted that the microseism amplitude in Antarctica strongly depends on the sea ice, which reduces the energy transfer from the hydrosphere to solid Earth (e.g., [33,34]). In particular, during the analyzed time intervals the Ross Sea showed a decreasing trend in both sea ice concentration and extent, reaching the minimum values in February 2023 (https://nsidc.org/data/seaice_index/compare-animate#anchor-2, accessed on 5 July 2023).

**Figure 11:**
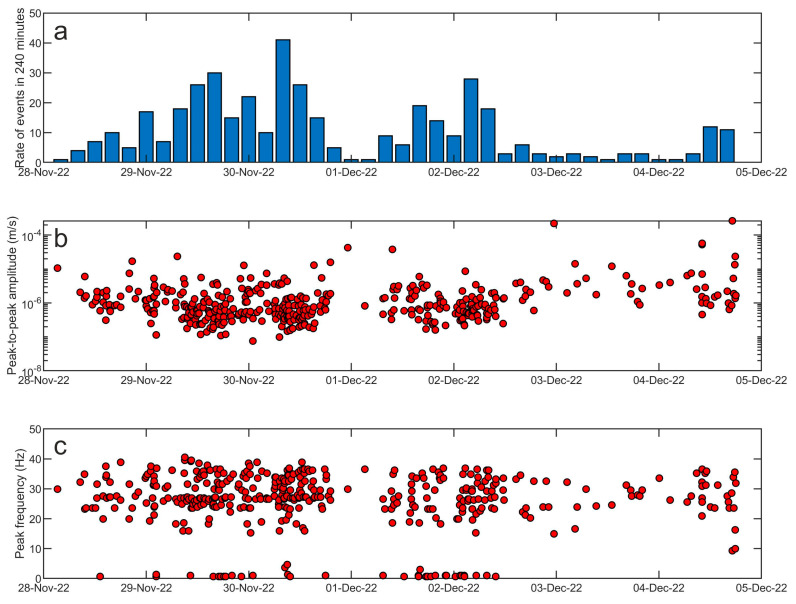
Features of the seismic events detected during the interval 28 November–4 December 2022 at the vertical component of the AURO station by short-time average/long-time average (STA/LTA; [23]). (**a**) Rate occurrence of seismic events in 4 h long time windows and corresponding (**b**) peak-to-peak amplitude and (**c**) frequency peak.

**Figure 12:**
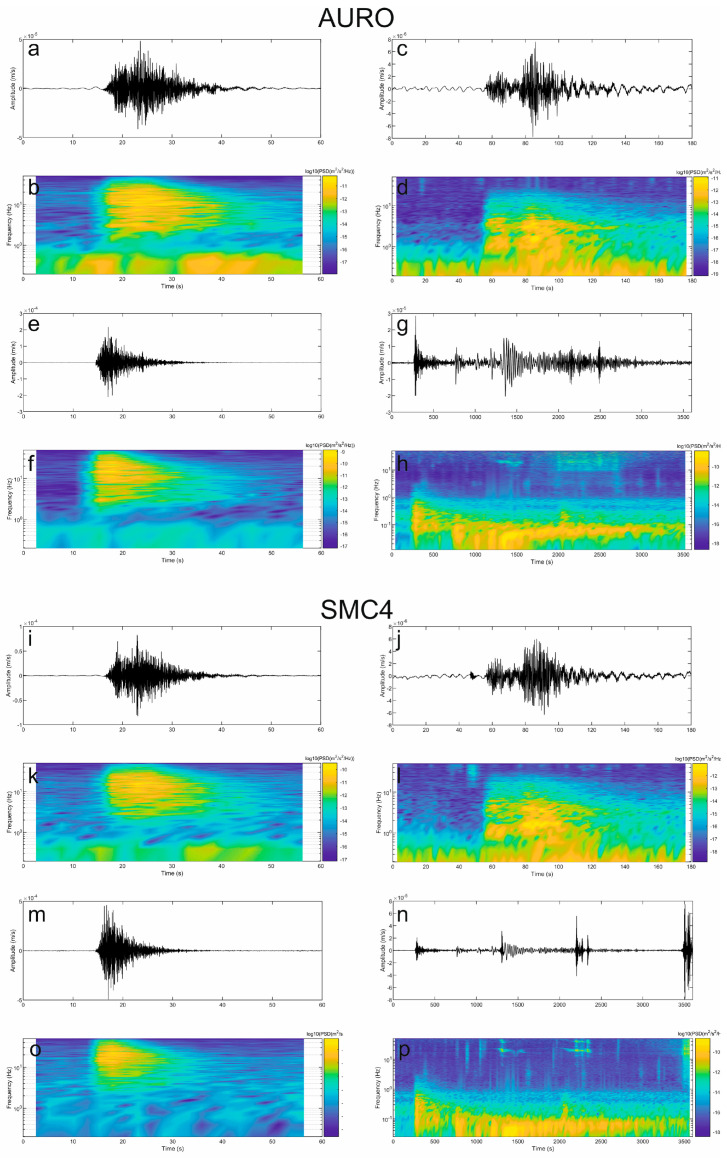
Examples of seismic event waveforms recorded by the vertical component of AURO and SMC4 stations, and corresponding short-time Fourier Transform (STFT). Seismic events recorded on 28 November 2022 at 04:15 (**a**,**b**,**i**,**k**), on 29 November 2022 at 13:13 (**c**,**d**,**j**,**l**) and on 2 December 2022 at 23:26 (**e**,**f**,**m**,**o**), and a teleseism recorded on 4 December 2022 at 19:30 (**g**,**h**,**n**,**p**). The signal shown in (**n**) and the corresponding STFT also exhibit high-frequency noise due to the ongoing installation and maintenance works in the MC4 ice cave during the teleseism. The STFT was performed by using 10.24 s long windows for the first three events (**b**,**d**,**f**,**k**,**l**,**o**) and 40.96 s long windows for the last event (**h**,**p**).

In terms of amplitude transients, a great variety of signals were collected during the analyzed period (Figure 11 and Figure 12). As for the rate of occurrence, it ranged from 0 to 10 events per hour. In terms of peak-to-peak amplitude and peak frequency, most values were in the range of 10^−7^–10^−5^ m/s and higher than 10 Hz, respectively. These events can have volcanic or ice origins. In the former case, they could be classified as high-frequency events and are likely associated with shear failure or slip on faults within the volcano edifice (e.g., [35] and references therein). Concerning the latter origin, a wide range of glacier-related processes are able to generate seismic signals with a very broad range of frequency content [36]. The investigation and discussion of the source of these events are beyond the scope of this paper and will be faced in future work. Also, teleseismic earthquakes are obviously recorded by these stations, as shown in Figure 12g,h,n,p, where the waveform of the earthquake that occurred at 19:24 on 4 December 2022 in the Tonga area with a magnitude of 6.8 (https://earthquake.usgs.gov/earthquakes/search/, accessed on 5 July 2023) is displayed. Finally, it is worth noting that long-lasting tremor-like signals with frequencies higher than 10 Hz are sometimes observed at both the stations (see in the interval 100–140 h in Figure 9b,d). Signals similar to these were also recorded in 2017 during an experiment performed at the summit of Mount Melbourne by using two temporary stations [10]. Due to the limited number of stations recording the signal, it is challenging to determine its exact source. In first approximation, two possibilities can be considered: (i) the movement of fluids within the volcanic plumbing system, in which case the tremor can be referred to as a volcanic tremor, and (ii) the discharge of water from beneath the glacier as a result of the heat released by volcanic activity in the fumarolic areas. Notably, similar tremor-like seismic signals associated with glacio-hydraulic processes have been observed in glacial regions recently (for instance, [37]). As mentioned for the amplitude transients, in the case of these tremor-like signals a detailed analysis and study are also beyond the scope of this paper and will be faced in future work.

**Figure 13:**
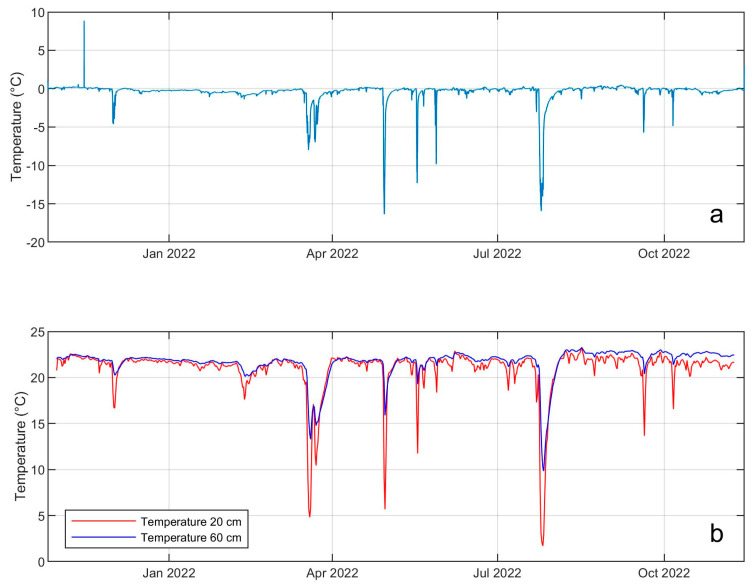
Example of temperature signals recorded by stand-alone air sensor installed near the geochemical stations with Tinytag Gemini device (**a**) and soil sensors at 2 different depths with Hobo devices (**b**) from October 2021 to October 2022 inside the Aurora FIC (see Figure 1).

**Figure 14:**
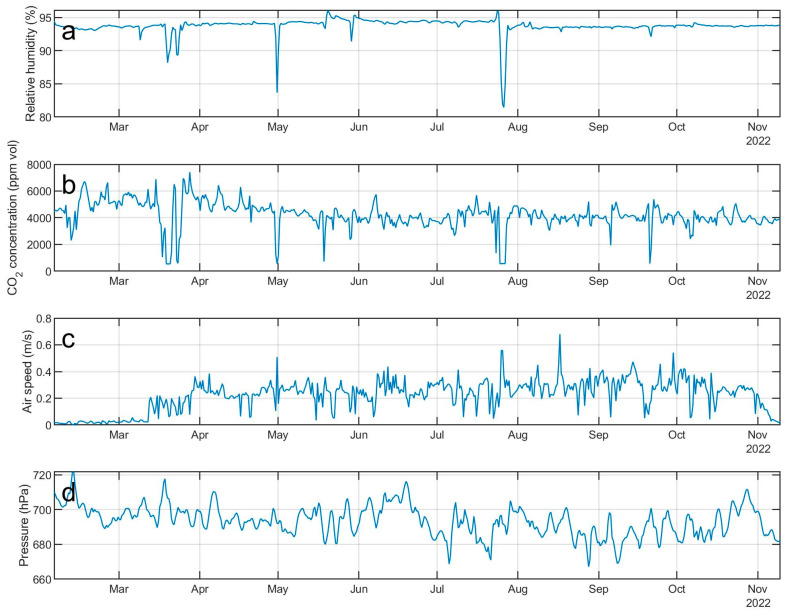
Time series of relative humidity (**a**), CO_2_ concentration (**b**), air speed (**c**) and pressure (**d**), recorded by the multi-gas station installed in the Aurora FIC during 2022.

Examples of geochemical and thermal signals, recorded by the Melbourne multiparametric network, are shown in Figure 13 and Figure 14. In particular, Figure 13 shows the temperature time series recorded by an air thermal sensor and two soil thermal sensors placed at two different depths from October 2021 to October 2022, exhibiting sharp decreases in temperature clearly recorded by all the three sensors. These thermal anomalies seem to be related to external atmospheric events, and not to variations in volcanic activity. Indeed, from preliminary investigations on data managed by the meteorological observatory of the MZS recorded by meteo stations located around Terranova Bay, katabatic winds or other strong atmospheric events were detected in the same periods as such sharp temperature decreases. Figure 14 shows the time series of parameters recorded by the multi-gas station installed in the Aurora FIC during 2022, and it is possible to note that such “anomalies” are also present in these signals. To date, the dynamics of these phenomena are not completely clear, but it is possible to state that during the anomalous periods we observed the following (Figure 13 and Figure 14): (i) a sharp decrease in temperature under the soil, both at 20 cm and 60 cm; (ii) a sharp decrease in temperature in the gas flowing inside the cave; (iii) a dilution of CO_2_ and H_2_O inside the cave. Pressure and airspeed, instead, seem not well correlated to the rest of recorded parameters. Further investigations will be carried out, and different deployments of the sensor system are under study, in terms of both geochemical and thermal sensors inside the ice caves and of a meteo station outside, to better understand the source dynamics beneath these anomalies and to develop a model explaining what really happens inside and outside the FICs in the case of strong atmospheric phenomena in the surrounding area.

**Figure 15:**
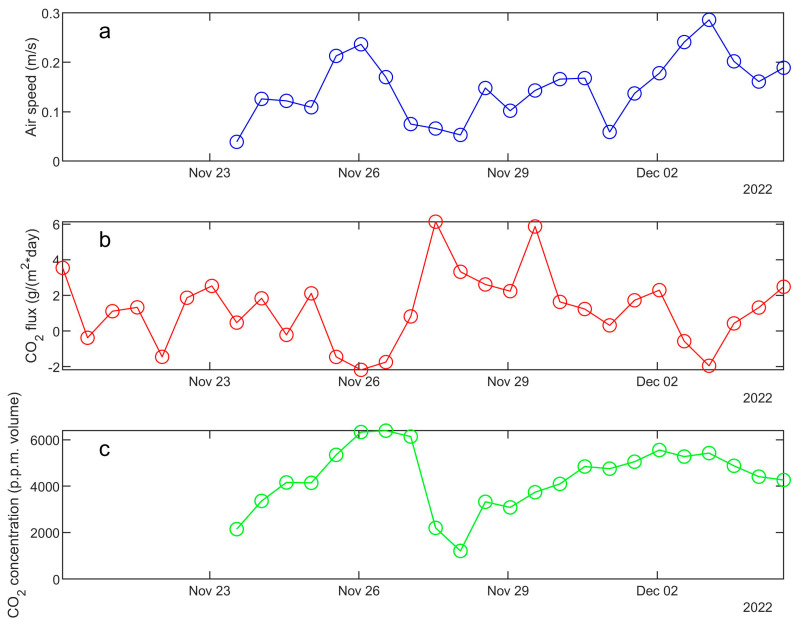
Time series of air speed (**a**), CO_2_ flux (**b**) and CO_2_ concentration (**c**) recorded by the multi-gas station installed in the Aurora FIC during 23 November–4 December 2022.

Figure 15 shows the time series of parameters recorded by the two geochemical stations installed in the Aurora FIC, multi-gas/meteo parameters from the MG2 station and CO_2_ soil fluxes from the automatic accumulation chamber. The stations were activated twice per day, and the data plotted are the result of post-elaboration of raw data collected in the server database. It is possible to note that in some brief periods the flux had negative values with high values of CO_2_ concentration in the atmosphere, while with relatively low values of concentration the soil flux was positive. Although the data presented here are just preliminary results (to the best of our knowledge, this is the first attempt to continuously monitor soil fluxes inside Antarctic FICs), it is important to underline that negative fluxes from soil are possible, as also reported in literature for other environments (e.g., [39,40]). This is likely due to the presence of soil areas with diverse contributions located along the tunnel and, hence, the concentration in a single section of the cave may change locally and temporarily due to external atmospheric perturbations. The ice cave itself acts as a gas integrator, where the relatively hot gas coming from different fumarolic areas flows from the bottom to the upper part, in a way similar to a chimney effect. In some areas, there is dilution with air coming from external parts, and all these contributions are affected by external meteorological conditions, even if on average the gas concentration along the cave increases from the bottom to the top. Thus, when the air speed through the cave increases, a dilution of gas concentration happens and the accumulation chamber reveals positive fluxes, because the concentration in the soil is higher than in the atmosphere. On the other hand, when the air speed is low the dilution is lower and the CO_2_ concentration in the air is higher than in the soil, so the gas diffuses from air to soil and the CO_2_ fluxes are negative.

## 5. Conclusions

Although most volcanoes in Antarctica are characterized by remoteness and inaccessibility, Antarctic volcanism is attracting the attention of the scientific community [41]. Indeed, recent records of eruptions from remote volcanoes (e.g., the 2010 Eyjafjallajökull eruption and the 2022 Hunga eruption) have strongly reminded us that even the farthest and/or least-known volcanoes can pose significant hazards to large and distant communities. In addition, the permanent settlement and seasonal presence of scientists, technicians, tourists and logistical personnel in Antarctica have increased significantly in the last decades. Hence, the need to create volcano monitoring facilities in Antarctica is becoming stronger and more urgent.

In this work, we describe the multiparametric monitoring system recently deployed on the Melbourne volcano, consisting of seismic, geochemical and thermal sensors together with powering, transmission and acquisition systems. Such a system has been designed to reliably work in polar regions, characterized by the most extreme climate on the planet in terms of very cold temperatures (reaching values lower than −40 °C) and extreme wind speeds (stronger than 120 km/h). FICs located on the summit area were chosen as installation sites since they are protected places where no storm can damage the instruments and temperatures are close to 0 °C all year round. The thermal conditions inside the FICs also allow to avoid the reduction in the battery capacity typical of very cold environmental conditions. An exception is the radio repeater, which was installed outside the FICs and thus is subjected to very cold temperatures; however, unlike the instruments inside the FICs, it has the possibility of exploiting solar energy for recharging during the Antarctic summers.

Particular strategies were used to power the stations and reduce the energy consumption: (i) a power system made up of large-energy-capacity accumulators, composed of rechargeable lithium batteries, was chosen; (ii) the seismic stations were set to “deploying normal” operating mode during October–February and “GPS and LAN Power Save” operating mode during February–October; (iii) the geochemical stations are activated just twice per day for about 30 min each time; (iv) the mini PC, installed in the Mario Zucchelli station and dealing with the data acquisition, was chosen based on its moderate current consumption.

In addition, the installations that were carried out on Mt. Melbourne did not require the creation of heavy infrastructures, such as surface vaults, disturbing the Antarctic environment. Indeed, fumarolic fields are specially protected areas, and hence the construction of a surface vault with infrastructures to guarantee insulation, power and resistance to the harsh environment (with the need of using poles, frames, shelters for instrument and so on) would disturb such an ambient environment. Inside the caves we use only mobile and “light” systems that are relatively easy to remove in case of necessity, with a low impact on the environment. Also, the reject batteries will be delivered to Italy, so the disposal of them will occur in Italy and not in Antarctica.

We demonstrate with our recent activity that continuous monitoring inside the FICs is feasible. In particular, variations in seismicity and geochemical and thermal data can suggest the onset of an unrest period. Also, heat flux variations can be computed by merging these data acquired inside the FICs with external meteo data. Thus, in the case of volcanic unrest (suggested by changes in seismicity and geochemical and/or thermal parameters), such a monitoring system can help issue warnings to the scientific stations located around the volcano. To date, data transmission and near-real-time analysis are possible only when the MZS is open (October–February). However, the stations also work during winter, collecting data that can be downloaded as the MZS opens. Further improvements are possible via the deployment of an external radio link powered by solar panels and wind generators so that a real continuous monitoring system, even during February-October, could be possible in the near future. Also, we plan to install additional seismic stations on the flank of the volcano, thus increasing the number of stations composing the seismic network and expanding such a network. This is a fundamental improvement in performing reliable location analyses of the detected seismic events.

Many challenges were faced during the creation of the network. Here, a few examples are shown. While deploying the instruments inside the caves did not represent a real challenge, apart from the difficulty to reach the chosen site and transport tools and devices inside the cave, sometimes using ropes and tools for rope progression, the installation of external radio repeater was more complicated. For instance, the first attempt to use radio repeaters powered by lead–acid batteries, even if of good quality, gave less than 20% of the expected autonomy. Hence, we decided to use LiFePO4 battery modules, and with the same weight we expected to obtain almost three times higher capacity; therefore, we significantly extended the autonomy of the device. During the test described in this paper, we obtained an autonomy of more than 90% than that expected in theory, despite the very low external temperatures (from −35 to −25 °C) that had severely reduced the lead–acid battery life. Using LiFePO4 instead of lead–acid batteries, we extended the autonomy by around ten times, with a similar total weight. Of course, the cost of the LiFePO4 modules was six times higher than the equivalent lead–acid batteries. Another challenge encountered was the monitoring of wind speed. We initially employed a couple of different ultrasonic sensors, but they failed to work after a few days, so we selected a heat wire anemometer, which has been working fine since 2019. Finally, we expected to encounter problems with the data transmission from inside the caves, due to that the roof of the cave is composed of ice and snow thicker than 10 m. However, surprisingly, with minimum power levels we were able to establish good radio connection to the server at the MZS.

The multiparametric monitoring system of Mt. Melbourne is acquiring a great amount of multidisciplinary data of interest for different scientific communities. Multidisciplinary data collected on this volcano will help to shed light on the volcano dynamics in polar environments, and this is especially precious as this volcano is one of the least known on Earth. For the geophysical community, seismic data can help better define the crustal and lithospheric structure in this area, as well as the recent crustal motions. For instance, seismic data recorded in this area were used to characterize crustal and tectonic structures in Victoria Land [42,43]. Glaciology and climate communities can use seismic data to extract information on glacier dynamics [36] as well as on the ocean state in terms of sea waves and sea ice concentration [34], helping to better characterize cryosphere and hydrosphere dynamics and their evolution over time. Concerning the former, it is worth noting that seismic data recorded in this area turned out to contain information on the dynamics of the David Glacier in terms of ice-quake generation [44], while for the latter the continuous acquisition of seismic signals in this area could help develop monitoring systems of the sea ice concentration, based on microseism elaboration, for northern Victoria Land, such as the one presented by [34]. In addition, the thermal data continuously acquired in the FICs could help the climate community to model the source of thermal anomalies identified so far, now under study [10]. Finally, the biological community can make use of the geochemical data collected inside the FICs to obtain information on fundamental microbe–mineral interactions contributing to the subsurface biosphere. Indeed, fumarolic ice caves house a dark oligotrophic volcanic ecosystem, offering a deep biosphere habitat that grants valuable knowledge about microbial communities harnessing energy sources beyond photosynthesis [45].

The technological strategies applied to develop the monitoring system of the Melbourne volcano and described in this work can help build efficient and reliable volcano monitoring infrastructures in polar environments.

## Data Availability

The data used in this work may be available upon request to the corresponding author.
